# A Practical Treatise on Diseases of the Skin, for the Use of Students and Practitioners

**Published:** 1883-06

**Authors:** 


**A Practical Treatise on Diseases of the Skin, for the use of Students and Practitioners.** By JAMES NEVINS HYDE, A. M., M. D., Professor of Skin and Venereal Diseases, Rush Medical College, Chicago, etc. Philadelphia: H. C. Lea's Son & Co. 1883.

There is, perhaps, no department of medicine which has, in this country, made greater advances than that of dermatology. Its didactic and clinical teaching has become a regular feature in all our leading colleges, and during the last decade many admirable text-books, colored plates and photographic portraits, have appeared from American authors. In view of this, it might reasonably be asked: What need of more books on this subject? After a careful review of Dr. Hyde's book, we must admit he has earned the right for its existence. Within a limit of 600 pages he has given a concise but really valuable treatise on cutaneous diseases, presenting, in a comprehensive form, the results of the latest observation and experience. The book is particularly adapted to the wants of the general practitioner, and we have no doubt will be as successful as its genuine merit deserves.

